# Supplementary material for: Hybrid seed incompatibility in Capsella is connected to chromatin condensation defects in the endosperm
Source: PLoS Genet. 2021 Feb 11;17(2):e1009370. doi: 10.1371/journal.pgen.1009370 (PMC7904229; doi:10.1371/journal.pgen.1009370)
Supplement: Attachment — Submitted filename: Response to reviewers.docx [file pgen.1009370.s009.docx]

Response to the editor`s and reviewers’ comments:

We would like to thank the editor and the reviewers for their overall positive evaluation of our work and suggestions for improvement. We revised the manuscript based on those suggestions and hope that you and the reviewers will find this revised manuscript suitable for publication.

Editor:

1. The hypothesis of limited maternal sRNAs being the cause of heterochromatin decondensation in hybrid seed endosperm is novel and interesting, as pointed out by Referee 2. Additional data supportive of this idea would significantly strengthen the paper. Ideally sRNA sequencing should be performed. However, we understand this might be difficult given the input requirement of sRNA sequencing and the difficulty of endosperm isolation, especially at this challenging time. Therefore, we propose to perform in-depth analysis of existing methylation data to strengthen the link between sRNAs, DNA hypomethylation and heterochromatin decondensation. The authors find TE CHG/CHH methylation reduced in the hybrid endosperm. Detailed analysis could be performed to examine the methylation reduction over heterochromatic vs euchromatic TEs, CMT3/2- vs RdDM- governed TEs, 24-nt sRNA enriched vs depleted TEs, TEs that are in the pericentromeric regions or on chromosome arms, etc.

These are interesting and relevant suggestions. However, due to the still incomplete annotation of the *Capsella rubella* genome, we currently lack the tools to perform those analyses. Out of the ~114000 TEs identified in the *Capsella* *rubella* genome, 105507 are short TEs and only 511 are longer than 4000bp (threshold use to define long TEs in Zemach et al. 2013). The large majority of the TEs that we found to lose DNA methylation in hybrid endosperm are short TEs, but given the annotation bias towards short TEs, this analysis is not appropriate to include in the manuscript. Considering only the annotated TEs, the data about their distribution (in pericentromeric and non-pericentromeric regions) are available in Supplementary table 1. In the previous version of the manuscript we included only the TEs that lose methylation in comparison to *Cr*, but now we updated the table with the TEs that lose methylation in comparison to both parents.

2. A novel link was made between gene misregulation and heterochromatin hypomethylation. Pheres1 target genes localized in the pericentromeric region were found preferentially upregulated in hybrid seed, which is interesting but not fully supported by the data. The misregulated genes were identified from Cr x Cr and Cr x Cg comparison, which may be influenced by genetic differences between Cr and Cg. Therefore, Cg x Cg endosperm transcriptome should also be included in the analysis. If available, data from reciprocal crosses should also be analyzed to narrow down the causal genes. This analysis might establish more clearly the preferential upregulation of pericentromeric Pheres1 targets (Line 233). Additionally, DNA methylation at AGL95 and 40 should be shown in the hybrid Cr x Cg endosperm and the Cr and Cg parental endosperm.

We consider the comparison *Cr* x *Cr* versus *Cr* x *Cg* as the relevant comparison, since the deregulation of genes in the *Cr* x *Cg* hybrid seeds is a consequence of loci transmitted by the *Cg* pollen. Thus, genes that have a different expression in *Cg* compared to *Cr* can be relevant and we would filter them out when comparing to both parents. For example; many type I AGLs are more strongly expressed in *Cg* compared to *Cr* (Rebernig et al., 2016); which is possibly relevant for the phenotype (since increased dosage of *PHE1* induces the triploid block in *Arabidopsis*¸ which corresponds to a phenocopy of the *Cr* x *Cg* seed phenotype). We also do not think that the reciprocal comparison to the cross *Cg* x *Cr* is relevant for this study; there is no good reason to assume that the same genes are causal in both type of crosses. We therefore think that the comparison *Cr* x *Cg* versus *Cr* x *Cr* is the most relevant one. Nevertheless, to address the editor’s concern, we have tested whether deregulated genes in comparison to both parents are also preferentially localized in pericentromeric regions, which is indeed the case (Figure S4A). We also found a significant overlap of PHE1 target orthologs with deregulated genes when comparing to both parents (Figure S4C).

We added the methylation analysis of the promoter region of *AGL95* and *AGL40* to Figure S5.

3. The authors find Cg contains more centromeric repeat copies than Cr, which, however, does not lead to CENH3 loading problems in hybrid seed. It is important to show that the mitotic problems are not related to the centromere and CENH3. However, the speculation (line 319-320) “that the cause for the [pericentromeric] hypomethylation is an imbalance of maternal Cr 24-nt sRNAs and Cg centromeric repeats” is confusing. How centromeric repeat number can influence pericentromeric heterochromatin is unclear. Furthermore, data on the specificity of the Capsella CENH3 antibody should be included.

Our data strongly argue that chromosome loss is not causal for the observed endosperm defects. We observe random chromosome loss in the endosperm, while the phenotype of seed abortion is similar among all aborted seeds, which strongly argues against that random chromosome loss and thus CENH3 mislocalization underpins the phenotype. Furthermore, we did not find defects in CENH3 loading (we repeated this experiment three times), also arguing against a causal role of CENH3. Lastly, the phenotype is endosperm specific; we did not find evidence for chromosome loss in seedlings derived from rescued embryos. Failure to localize CENH3 would be difficult to reconcile with an endosperm-specific defect.

We think that indeed changes in centromere repeat number may impact on pericentromeric chromatin condensation. Centromeric repeats in *Arabidopsis* are also present in pericentromeric regions, where they are heavily methylated (Zhang et al. 2008). Thus, increased numbers of centromeric repeats may impact on methylation levels outside of the centromere. We included this point into the discussion.

We found co-localization of CenH3 with the chromocenters in the nuclei, as expected for a specific CENH3 recognizing antibody. Furthermore, the peptides recognized by our antibody differ between H3.1/H3.3 and CENH3, making it unlikely that the antibody binds unspecifically to H3. We added this alignment into Supplemental Fig 2.

Reviewer's Responses to Questions

Reviewer #1:

The manuscript by Lauriane Simon et al provides an interesting perspective on the way endosperm development, but not embryo development induces mitotic abnormalities. They link this pattern of development to defaults to both CHG and CHH methylation, and suggest that PHE1 which acts as a transcription factor, might play an important role in the hyper activation of several relevant targets within pericentromeric heterochromatin. While the manuscript is well written and quite interesting, it suffer from a number of limitations that would need to be addressed to validate the core conclusions.

* importantly, the authors need to clarify how many replicates were performed for the chromosome spread in Fig 2A, B and C. I have not seen any numbers reported in the Material and Methods.

The experiment has been performed in three independent replicates. We added this information to the Methods.

* Similarly, I was a bit confused with the number of replicates generated for the BS-seq analyses. While it may be difficult to generate enough samples from very early endosperm, the authors should provide the number of replicates generated. That would strengthen their argument.

We generated two biological replicates and added this information to the methods.

* I’m also a bit confused with the fact that they did not observed any differences in the methylation level in genes (Fig S3), but report statistically significant differences on S3-B. How does that fit with the expected pattern ? I might have missed something, but I find it confusing.

We agree and changed the sentence to “…while genes had an intermediate level of DNA methylation in all sequence contexts compared to the parental species.”

* Surprisiny, CENH3 does not seem to have much impact of the compaction of chromocenters. Have the authors looked more closely, with enough replicates, to have enough certainty that CENH3 indeed does not account for chromosome loss ?

We performed the experiment in three replicates and did not observe an obvious deviation in CenH3 localization. We therefore consider a causal role of CenH3 in this process as rather unlikely. Also given that the chromosome loss is specific for the endosperm and not observed in the embryo makes in our view a causal role of CenH3 unlikely.

* While the data on TEs is quite interesting, Wilcoxon’s test most likely over-estimates the actual differences, the spread being rather broad. Student-test might be more appropriate.

Since the data are not normally distributed, which is a precondition for a Student’s t-test, we think that the Wilcoxon test is the suitable test for this analysis.

* Also quite important, I could not understand how the DNA methylation analyses were performed on the PHE1 targets orthologs. While the promoter region is clearly important for DNA methylation, I could not really understand how the significance was calculated, and how the authors compared the PC versus NonPC targets. Did they average the mean in the CG, CHG and CHH context? This needs to be clarified in the discussion, possibly adding the methodology used to compute the statistical tests.

We compared the average level of DNA methylation in the 500bp upstream region of the transcriptional start site of PC and NPC PHERES’ target orthologues and performed a Wilcoxon test for statistical significance. We added this information to the figure legend.

minor point:

line 125 «Hybrid endosperm shows mitotic problems and random chromosome loss » should read « Hybrid endosperm shows mitotic abnormalities and random chromosome loss »

Done.

Reviewer #2:

Review of ms entitled “Hybrid seed incompatibility in Capsella is connected to chromatin condensation defects in the endosperm” by Dziasek et al.

The work presented here seeks to better understand the poorly understood underlying mechanisms provoking seed abortion during hybridization of two recently diverged brassicaceae species.

Authors perform reciprocal crosses between Capsella rubella (referred to as Cr)  and Capsella grandiflora (referred to as Cg) and focus on studying the fate of the endosperm in the resulting hybrid seeds. Indeed, it is known that although both species have the same ploidy level, they are separated by a strong endosperm-based hybridization barrier (i.e. seed development is in part strongly perturbed as a result of poor endosperm development).

A QTL analysis identified three QTLs associated with Cr x Cg incompatibility. These QTLs are localized in pericentromeric regions, i.e. regions of condensed chromatin. No specific genes where identified, rather extended pericentromeric DNA regions.

Furthermore, authors found that in hybrid seeds:

1) the hybrid endosperm has mitotic problems and displays random chromosome loss. Authors convincingly show that chromosome loss is not attributable to the centromere-specific variant of H3 (CENH3).

2) the chromatin in the hybrid endosperm is decondensed, which is associated with loss of DNA methylation.

3) deregulated genes in hybrid endosperm are mostly localized in pericentromeric regions, in particular authors found increased expression of PHERES1, encoding a TF previously shown to account for triploid seed arrest.

The finding that hybrid endosperm displays random chromosome loss and lower DNA methylation levels was already reported previously (references are provided by the authors).

Previous studies that we cited in the manuscript reported uniparental genome elimination in embryo and endosperm of *H. vulgare* x *H. bulbosum* hybrid seeds. This was found to be due to specific failure of CenH3 loading to the *H. bulbosum* genome, which consequently gets eliminated. This is distinct to the random loss of a single chromosome in the endosperm, as observed in our study.

Furthermore, previous reports showed (using A. lyrata and A. thaliana) that hybridization can cause chromosome chromatin compaction defects (e.g. Zhu et al Genome Biology 2017). So in this regard the findings reported in this work are not substantially novel.

The study by Zhu and colleagues focused on the sporophyte generation, not the endosperm. Furthermore, Zhu et al. excluded the pericentromeric regions from the analysis. Moreover, the effect they observed is different to what we found, since they report that the chromatin compactness in the hybrid is intermediate between two parents, while we found that the pericentromeric regions are decondensed in comparison to both parents. Therefore, what we report in our study has not been previously published and is therefore novel.

The novelty present in this report could be the proposition that lower DNA methylation (inducing decondensation and chromosome loss) is due to lower sRNAs provided by maternal tissues as a result of their lower pericentromic repeat copy number. But this was not tested. Authors propose that seed abortion is due to high PHERES1 expression but it might just as well be due the result of mitotic problems and/or chromosome loss.

Our data strongly argue that chromosome loss is not causal for the observed endosperm defects. We observe random chromosome loss in the endosperm, while the phenotype of seed abortion is similar among all aborted seeds, which strongly argues against that random chromosome loss underpins the phenotype. We furthermore find a strong enrichment of PHE target genes being deregulated in the hybrid seeds, which is specifically pronounced in pericentromeric regions. Given the phenotypic and molecular similarities of triploid Arabidopsis seeds and Capsella hybrid seeds (Rebernig et al., 2016) and the fact that PHE1 is causally responsible for triploid seed abortion in Arabidopsis (Batista et al., 2019), supports the idea that rather deregulated PHE1 targets than chromosome loss underpins the phenotype. Furthermore, we found three QTL to connect to hybrid seed arrest, which would also be difficult to reconcile with loss of a single chromosome being causal for the phenotype.

As mentioned above, similar observations were done using crosses involving parents of different ploidy levels. In these cases, authors proposed different mechanisms accounting for seed abortion that include ideas for mechanisms similar to those proposed here (e.g. Batista et al 2019, Erdmann et al 2017) but also other ones (e.g. Dilkes et al. 2008). However, they were tested by genetic means.

As mentioned above, the phenotypic similarities of triploid Arabidopsis seeds and hybrid Capsella seeds points to a common genetic mechanism underlying both phenotypes. In Arabidopsis, mutants in PHE1 suppress the triploid block (Batista et al., 2019). Since orthologs of PHE1 are deregulated in Capsella hybrid seeds and similarly, PHE target orthologs are deregulated in Capsella hybrid seeds, we propose that also this deregulation of PHE1 orthologs is causal for the Capsella hybrid seed arrest. Genetically testing this hypothesis, however, is a highly time-demanding undertaking, since there are several potential PHE1 orthologs that may be responsible. Identifying the causal one will take substantial amount of time and effort and would go beyond the scope of this manuscript. The study by Dilkes at al. the reviewer is referring to identified TTG2 as a maternal suppressor of the triploid block. In our study, we focused on the paternally-responsible genes contributed by the Cg parent and their effect in hybrid seeds, which is unrelated to the work by Dilkes et al. Likewise, Erdmann et al., 2017 and also our own work (Martinez et al., 2018) revealed a role of paternally-contributed small RNAs in establishing the triploid block and we proposed that increased dosage of PolIV-dependent small RNA contributed by pollen is responsible for hypomethylation in the endosperm of triploid seeds (Martinez et al., 2018). In this study we show that like in triploid seeds, Capsella hybrid seeds have hypomethylated endosperm, which may in combination with deregulated PHE1 orthologs connects to the observed phenotype. This idea has been discussed in the manuscript.

Reviewer #3:

In this manuscript from the Dziasek et al, the Köhler lab continue their work on the effects of hybridization on endosperm development and seed viability in Capsella species. Using genetic, epigenetic, genomic, and cytological tools, the authors show that crosses of C. rubella with C. grandiflora show problems with mitosis and chromosome conservation in the endosperm, that the endosperm in these hybrids has reduced cytosine methylation and chromatin condensation, and that many PHERES1 target genes in pericentromeric regions are upregulated (likely the explanation for endosperm arrest). The authors also identify 3 QTLs, located in pericentromeric regions, which are responsible for endosperm arrest in Cr x Cg crosses. And the authors present correlative evidence that the effect Cr x Cg crosses on the endosperm, similar to the effect of 2x x 4x crosses in Arabidopsis, is due to an increase in pericentromeric repeat regions in Cg compared to Cr.

I have only minor comments:

lines 29-30: the can't say that the embryo was not affected, because they weren't able to judge the phenotype of chromatin in the embryo, because it was less dense in the parents of the hybrid. They should remove the reference to the embryo in this sentence.

We agree that we cannot directly conclude that chromatin condensation in the embryo is not affected; however, since we did not observe chromosome loss in the hybrid, it seems likely that chromatin condensation is normal. Nevertheless, we removed this statement.

lines 32-34: The mention of 'chromosome loss' comes out of nowhere, and needs to be better explained. In the previous sentence they talked about loss of chromosome condensation, a different effect of hybridization, making this sentence doubly confusing.

We agree, we rephrased the previous sentence to make the connection.

A general comment about the abstract: it's not as well written as the author summary, which is very nice.

Thanks for this comment. We rephrased the abstract, hoping it reads better now.

line 67: 'syncitium': it's more correct to refer to the endosperm as a coenocyte. for definitions see: <https://en.wikipedia.org/wiki/Coenocyte>

Agreed, done.

line 88: I think this should be '2x x 4x', not '4x x 2x'

Thanks, done.

lines 143-144: the authors should modify this sentence. They didn't show that chromatin decondensation and chromosome loss were restricted to the endosperm. They showed that these things occur in the endosperm, and not in the root. There were not able to quantify the embryo. And they didn't look at any other tissues (not necessary look at other tissues), but they should modify the sentence to say the chromosome effects don't occur in all tissues, or something like that.

We removed the reference to the chromatin condensation and only kept the statement about the chromosome loss that is specific to the endosperm, since we did not detect it in root tissue. If chromosome loss would occur in the embryo, we would have detected it in the roots of the plants derived from rescued embryos.

lines 181-183: this sentence has the first phrase in the present tense (are), and the next phrase in past tense (contained).

Done

line 209: losing is misspelled as 'loosing' (also in S1 Table).

Done

line 216: 'were' instead of 'was'

Done

line 299: 'are' instead of 'is'

Done

line 319-320: The sentence 'We speculate that the cause for the hypomethylation is an imbalance of maternal Cr 24-nt sRNAs and Cg centromeric repeats.' is very interesting, and provides a possible mechanistic explanation for the effect on the endosperm that they see. I think it would be good to include a sentence like this is the abstract, because it ties all of their results together.

We are happy that the reviewer appreciates this idea, but since this remains an idea and remains to be thoroughly tested, we think it would not be appropriate to move this part to the abstract.
